# Insight into Structural Changes and Electrochemical Properties of Spark Plasma Sintered Nanostructured Ferritic and Austenitic Stainless Steels

**DOI:** 10.3390/nano12071225

**Published:** 2022-04-05

**Authors:** Junaid Ahmed, Ihsan-ul-Haq Toor, Mohamed A. Hussein, Nasser Al-Aqeeli, Mirza M. A. Baig

**Affiliations:** 1 Department of Mechanical Engineering, King Fahd University of Petroleum & Minerals (KFUPM), Dhahran 31261, Saudi Arabia; jahmed9@uic.edu (J.A.); mmurtuza@kfupm.edu.sa (M.M.A.B.); 2 2095 Engineering Research Facility, Department of Civil, Materials and Environmental Engineering, University of Illinois at Chicago, Chicago, IL 60607-7023, USA; 3 Interdisciplinary Research Center for Advanced Materials, King Fahd University of Petroleum & Minerals (KFUPM), Dhahran 31261, Saudi Arabia; mahussein@kfupm.edu.sa; 4 Department of Mechanical Engineering, King Saud University (KSU), Riyadh 11421, Saudi Arabia; naqeeli@moe.gov.sa

**Keywords:** nanostructured Fe-Cr alloy, mechanical alloying, spark plasma sintering, % densification, electrochemical testing, corrosion mechanism

## Abstract

Nanostructured ferritic (Fe_(82−x)_-Cr_18_-Si_x_, x = 0–3 wt %) and austenitic (Fe_(73−x)_-Cr_18_-Ni_9_-Six, x = 0–3 wt %) stainless steel (SS) alloys were developed by mechanical alloying (MA) and spark plasma sintering (SPS). The unit cell parameter estimated from X-ray diffraction spectra exhibited a decreasing trend with an increase in wt % of Si content in both alloy systems. The particle size of powders estimated using bright field transmission electron microscopy images for ferritic (3 wt % Si) and austenitic (3 wt % Si) SS powders was found to be 65 ± 5 nm and 18 ± 3 nm, respectively. In case of the ferritic system, 3 wt % Si exhibited the highest densification (~98%) and micro-hardness of about 350.6 ± 11.2 HV, respectively. Similarly, for the austenitic system (3 wt % Si), maximum densification and micro-hardness values were about 99% and 476.6 ± 15.2 HV, respectively. Comparative analysis of potentiodynamic polarization, linear polarization, and electrochemical impedance spectroscopy results indicates an increase in electrochemical performance of both alloy systems as the wt % Si was increased. The increase in electrochemical performance is directly related to the increase in densification owing to Si addition in these alloys.

## 1. Introduction

Stainless steels (SSs) are the most diversely employed Fe-based alloys—finding their applications in oil and gas, power generation setups, desalination plants, nuclear reactors etc.—owing to their excellent corrosion resistance and mechanical properties. The development of powder metallurgy (PM) has revolutionized the near-net shape bulk synthesis of complex geometries with a variety of metallic powders [1]. SS components synthesized via PM constitute a major and growing segment of the PM industry [2,3,4]. Amongst the SS families, austenitic SSs exhibit superior mechanical and electrochemical properties [5] and find their applications in automotive, marine, food, biomedical industry, and high-temperature applications [6]. However, synthesis of PM ferritic SSs components is gaining popularity due to their lower production cost, considerable corrosion resistance [7,8,9], and mechanical properties [10], and they are frequently used in automotive exhaust systems, containers [11], solid oxide fuel cells [6], etc.

It is generally believed that powder metallurgy (PM) alloys exhibit lower corrosion and oxidation resistance as compared to wrought alloys of similar chemical compositions [12,13,14]. There are many reasons for this poor performance; however, porosity is the main reason, as it is challenging to produce 100% dense (free of porosity) materials by this processing technique. Regarding corrosion, performance of a SS depends heavily on the quality of its protective film, so the presence of porosity can result in poor quality films that will decrease the corrosion resistance of these alloys [15]. Controlling the percentage of porosity in PMs part is critical in improving their mechanical and corrosion properties. The most leveraged method to improve the density of PM components is optimizing the sintering conditions; particularly, sintering temperature and holding time. Toor et al. systemically optimized the sintering conditions of ball-milled Fe-18Cr-2Si powders [16]. It was reported that with the increase in sintering temperature and holding time of sintering, densification increases linearly. Besides temperature and time, sintering atmosphere also affects the overall densification of SS powder as reported by Mariappan et al. [17]. Garcia et al. [18] highlighted the benefits of sintering in vacuum in comparison to nitrogen and hydrogen. Similarly, Martin et al. [11] reported the effect of slower cooling rates in increasing the density of the alloys. Furthermore, Ozturk et al. [6] reported an increase in densification with an increase in compaction pressure. Along with optimization of sintering parameters for maximum densification, some alloying additions are reported to enhance the overall compaction of sintered specimens. It is reported that the addition of copper [19], boron [20], etc., in SS powders improves their densification. There are some reported studies claiming an increase in corrosion resistance of nano-crystalline PM parts in comparison to micro-grained counterparts [21,22]. However, there are not many systematic studies evaluating the effect of minor alloying additions on the densification—and consequently, corrosion—performance of SPS SSs.

Among the different available routes of preparing nano-crystalline alloy powders, mechanical alloying (MA) is considered one of the promising techniques for such applications [23,24,25,26]. This technique involves solid-state reactions to synthesize equilibrium and non-equilibrium structures including supersaturated, metastable crystalline, quasi-crystalline, intermetallic, nanostructured, and amorphous alloys [27,28]. The succession of fracture and welding due to milling action leads to the formation of nanometer-sized particles [27]. Sintering of nano-crystalline alloy powders is crucial in salvaging the nanostructure due to possible grain growth during the sintering process. Conventional sintering is employed widely; however, due to intrinsically slower heating rate, longer holding times (usually in h), and slower cooling rates, it results in grain growth. Spark plasma sintering (SPS) is a relatively new and novel technique utilizing a simultaneous application of pressure and pulsed continuous current (AC) to consolidate the powders [29]. The time required to consolidate powders using the SPS technique is much shorter in comparison to conventional sintering techniques. Furthermore, faster cooling rates in conjunction with shorter sintering time results in retention of the nano-crystalline structure.

The commercially available SSs do have a small amount of Si and the main reason for such an addition is oxygen absorption along with an increased oxidation resistance of such alloys, irrespective of ferritic and austenitic SSs [30,31,32,33,34,35,36,37]. However, it can also result in the degradation of corrosion resistance [38] due to the formation of secondary phases. The increase in corrosion resistance can be attributed to the presence of Si in the passive film. Riffard et al. [39] suggested the formation of an SiO_2_ layer at the interface of outer Cr_2_O_3_ (passive film) and the base metal, thus providing additional protection to the base metal. Although there are few studies on the mechanical alloying and corrosion performance of Fe-Cr based alloys [13,22], the independent effect of Si on densification and corrosion performance of nanostructured austenitic (Fe_(73−x)_-Cr_18_-Ni_9_-Si_x_, x = 0, 1, 2, 3 wt %) and ferritic (Fe_(82−x)_-Cr_18_-Si_x_, x = 0, 1, 2, 3 wt %) alloys prepared by MA and SPS is not well documented in the literature. Therefore, the main objective of this research work was to synthesize nano-structured austenitic and ferritic stainless steel powders with varying Si content via mechanical alloying, followed by SPS and the conducting of an in-depth study on their electrochemical performance. The presence of nanostructure will help in achieving better densification and so will improve the corrosion resistance of the alloys along with the presence of Si in these alloys.

## 2. Materials and Methods

High purity (>99.99%) Fe, Cr, Ni, and Si powders—with particle sizes of <45 μm, <140 μm, <100 μm, and <50 μm respectively—were used to prepare the nominal compositions of Fe_(73−x)_-Cr_18_-Ni_9_-Si_x_ (x = 0, 1, 2, 3 wt %—γ) and Fe_(82−x)_-Cr_18_-Si_x_ (x = 0, 1, 2, 3 wt %—α) alloys. Ferritic powders were milled for 100 h and austenitic powders for 160 h respectively and the details are reported elsewhere [40]. Powders were mechanically alloyed (at room temperature) in a planetary ball mill (Fritsch Pulverisette 5, FRITSCH GmbH - Milling and Sizing, Weimar, Germany) for 5, 10, 15, 20, 35, 65, 85, 90, and 100 h (for ferritic SS) and extended to 160 h for austenitic SS powders respectively. The powder mixtures were ball milled in SS vials (250 mL) with 10 mm SS balls in an argon environment, with a ball-to-powder ratio (BPR) of 30:1. The rotating speed was set to 300 rpm and to avoid cold welding and agglomeration, only 3% stearic acid was utilized. After every 1 h, the milling was interrupted for 30 min to dissipate any heat energy and to bring the vials to room temperature. Austenitic SS powders (after 160 h milling) were subsequently annealed at 1100 °C for 1 h under an Ar atmosphere.

After mechanical alloying, ball-milled powders were sintered in an automated, spark plasma sintering machine (HP D-5, FCT Systeme, Frankenblick, Germany). A 20 mm graphite die (current was passed through it) was used to hold the powders. The alloys were sintered in vacuum to avoid oxidation and a thermocouple was used for temperature measurement. To ease the specimen removal once the sintering process was completed, a sheet of graphite was employed which also helped in reducing the friction issues between the die and powder. Sintering was performed at 1100 °C for 15 min with an applied pressure of 60 MPa and a heating rate of 100 °C/min under vacuum and sintering parameter optimization (sintering time, temperature, pressure, and heating rate) is explained elsewhere [16]. Sintered samples were metallographically cleaned before measuring the density and Vickers hardness. After the sintering of the alloys, the density was measured based on Archimedes’ principle and a digital Vickers hardness tester (Buehler, Lake Bluff, IL, USA) was used for measuring the hardness of the developed alloys. While measuring the hardness, a load of 500 g was used with a dwell time of 10 s. For accuracy and repeatability, an average of 10 data readings—both for density and hardness—is presented. Phase identifications of ball-milled powders and sintered specimens were studied by using an XRD, Bruker D8 advance equipped with Cu Kα radiation (λ = 0.142 nm). XRD was performed over a 2θ range of 30–95° with an angular increment of 0.05°. Unit cell parameter (a) of ball-milled powders was calculated by determining the d-spacing of highest Bragg’s angle and implementing the equation

$$a=d*\;\sqrt{h^{2}+k^{2}+l^{2}}$$

A JOEL field emission scanning electron microscope (FE-SEM) equipped with energy dispersive spectroscopy (EDX) was leveraged to confirm the chemical composition of ball-milled powders as well as after sintering. A JOEL FE-transmission electron microscope (FE-TEM) was utilized to confirm the XRD data collected from MA powders. For TEM microscopy, the powders were dispersed in acetone in a sealed glass container and ultrasonicated for 5 min to remove their stickiness. For particle size analysis of milled powders, low magnification TEM images were taken, and the average of at least 20 particles is reported here.

A three-electrode corrosion cell (working electrode, platinum auxiliary electrode, and a saturated calomel reference electrode) was used for electrochemical tests. Grinding was carried out up to 2000 grade of SiC abrasive paper before applying 0.5 μm alumina suspension for mirror polishing. Finally, the samples were ultrasonically cleaned in acetone before performing the corrosion experiments. For corrosion tests, an exposed surface area of 0.1 cm^2^ was selected for accuracy and repeatability, and experiments were performed in 0.2 M NaCl solution at room temperature. A Gamry Reference 3000 potentiostat was used for these experiments, which were repeated thrice to confirm the authenticity of the reported data. A scan rate of 0.2 mV/s was selected for potentiodynamic polarization (PDP) experiments using DC105 software, which was subsequently used for data analysis as well. The common practice of cathodic cleaning (for removing any traces of surface oxides) was employed at −1.0 V_SCE_ for 180 s, before corrosion experiments. A sinusoidal voltage perturbation signal of 10 mV_SCE_ with a frequency range of 100,000 to 0.2 Hz, was used for electrochemical impedance spectroscopy (EIS) tests. To evaluate the electrochemical impedance response of the alloys, first an oxide film on the surface of these alloys. The potential selected for this film formation was 0.05 V_SCE_ in deaerated 0.2 M NaCl solution, based on PDP data. The duration for film formation was 30 min under potentiostatic conditions. Linear polarization resistance (LPR) experiments were carried out over a range of potentials (±20 mV_SCE_) around the open circuit potential (OCP) using a scan rate of 0.3 mV/s. Before performing the LPR experiments versus open circuit potential (OCP), its value was measured, after the samples were kept inside the solution for 30 min.

## 3. Results

### 3.1. Characterization of Ball-Milled Powders

**Figure 1:**
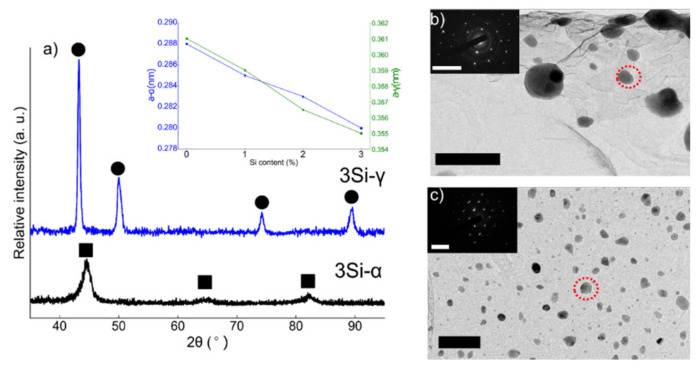
(**a**) XRD spectra obtained for ferritic (Fe_(82−x)_-Cr_18_-Si_x_, x = 3 wt %) and austenitic (Fe_(73−x)_-Cr_18_-Ni_9_-Si_x_, x = 3 wt %) stainless steel powders after ball milling at 100 h and 160 h + annealing, respectively. Ferritic SS powders are represented as γ and austenitic SS powders with γ. The square markings in (**a**) represent the BCC peaks and the circles denote FCC peaks. The inset in Figure 1a represents a change in unit cell constant with the change in % Si content. With the increase in Si content in the alloy powder, the lattice parameter decreases monotonically. (**b**,**c**) Bright field TEM images of ferritic (3Si) and austenitic (3Si) powders and the insets are the selected area diffraction patterns obtained from the areas encircled in red. The scale bars in (**b**,**c**) represent the length of 100 nm and 50 nm, respectively and the reciprocal space scale bars in the insets are 10 1/nm.

XRD spectra collected from ferritic (Fe_(82−x)_-Cr_18_-Si_x_, x = 3 wt %) SS powders milled for 100 h and austenitic (Fe_(73−x)_-Cr_18_-Ni_9_-Si_x_, x = 3 wt %) SS powders milled for 160 h followed by annealing is presented in Figure 1a. Examination of the XRD spectra confirms the BCC crystal structure of 3 wt % Si ferritic SS powders and FCC microstructure of austenitic powders for all the compositions. The inset in Figure 1a represents the evolution of the lattice parameter with the addition of % Si content in both ferritic and austenitic SS powders. There was a monotonic decrease in the unit cell parameter with the addition of Si and this trend is in complete agreement with the data published previously [40]. Figure 1b,c are the bright field (BF) TEM images collected from 3Si-100 h milled ferritic SS powders and 3Si-160 h milled + annealed austenitic SS powders. It is obvious from the BF-TEM images that the austenitic powders are much finer than ferritic powders due to extended milling. The insets in Figure 1b,c are the selected area diffraction (SAD) patterns collected from the areas encircled in red-dotted circles. The SAD patterns confirm BCC structure for the ferritic powders and FCC structure for austenitic powders as reported above based on XRD results. To confirm the particle size of the powders after milling, low-magnification BF-TEM images were taken at various locations and the average particle size is reported here. The average particle size of ferritic (3 wt % Si) and austenitic (3 wt % Si) SS powders was 65 ± 5 nm and 18 ± 3 nm respectively.

**Figure 2:**
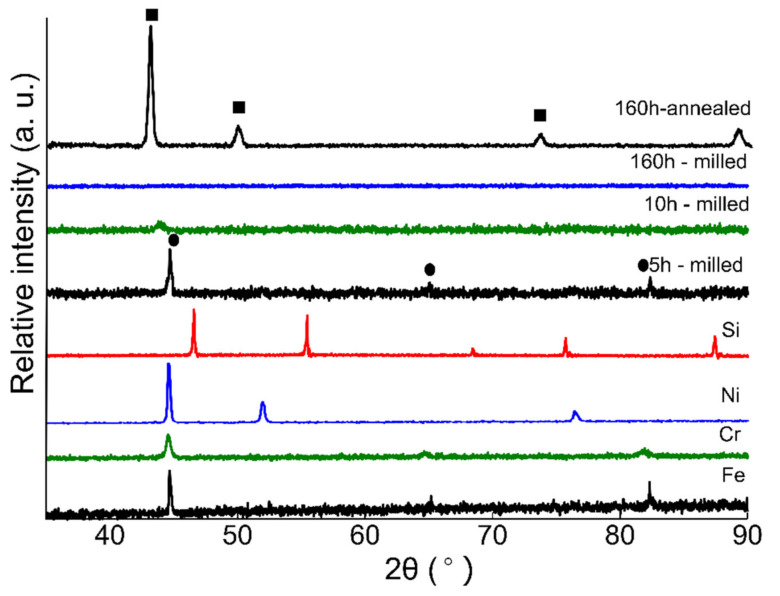
XRD spectra collected from ball-milled austenitic SS powders (Fe_(73−x)_-Cr_18_-Ni_9_-Si_x_, x = 2 wt %) at various milling times. XRD spectra collected from pure elements are also presented. The spectrum obtained from 5 h milled sample exhibits a BCC structure (indicated by circular markings). Continued milling resulted in amorphization of milled powders. The spectra collected from 20–150 h milled powders are not presented here. The 160 h milling sample spectrum exhibits an amorphous structure. The spectrum collected from 160 h annealed powder exhibits an FCC structure and the peaks are represented by square markings.

Figure 2 presents the XRD spectra collected from powder specimens with nominal composition Fe_(73−x)_-Cr_18_-Ni_9_-Si_x_ (x = 2) milled for 5, 10, 160 h, and the 160 h annealed sample presents phase evolution during ball milling. XRD spectra from pure Fe, Cr, Si, and Ni powder are also presented before milling. The first specimen was collected after 5 h of ball milling and the XRD spectrum is presented in Figure 2. The microstructural investigations reveal that ball milling resulted in solid solution formation and the alloyed powders exhibit a BCC crystal structure. Continued milling to 10 h resulted in considerable peak broadening, as well as a peak shift toward smaller angels representing the increase in lattice parameter due to alloying. Crystallite size refinement and lattice strains developed due to milling action can result in peak broadening in the XRD pattern as reported by Patil et al. [41]. Even though Ni is a strong austenite stabilizer, the formation of α-phase rather than γ-phase at 5 h of milling is not unexpected. Kaloshkin et al. reported the formation of an α-solid solution at early stages of mechanical alloying of Fe-Ni systems with varying Ni content. The authors highlighted that continued milling of α-phase powders resulted in the formation of γ-solid solutions, which was confirmed by XRD pattern [42]. Similarly, Enayati et al. [43] observed the same phase evolution of stainless steel powders and the α-phase transformed into γ-solid solution after 60 h of milling. Similar phase evolution was reported by Oleszak et al. [44] and they obtained γ-solid solution after milling for 100 h. These studies [42,43,44] indicate that the formation of α-phase and γ-solid solution depends upon milling time. The XRD spectrum collected from the 20 h milled specimens did not show any visible peak (not presented here). Various authors [26,27,40,41,45,46] have reported this type of behavior—vanishing of XRD peaks after longer milling time—and attributed the phenomenon to the loss of long-range order (crystalline structure). Suryanarayana et al. [27,46] reported that with the progression of milling, the long-range order of constituent particles is broken down to a short-range order because of the formation of different crystal defects such as dislocations and vacancies due to severe plastic deformation. Movahedi et al. [45] investigated the Fe–Cr–Mo–B–P–Si–C system and observed the formation of saturated solid solution at early stages of milling which later with continued milling transformed to an amorphous phase. Similarly, Sharma et al. [26] also reported the vanishing of Fe(110) peaks at 10 h of milling due to amorphous phase formation. Falcao [47] reported the difference in the atomic sizes of the constituent elements and that the lattice strain generated due to milling action can result in the formation of an amorphous phase during MA of metallic powders.

The XRD spectrum collected from 160 h milled powder indicates that the powders are still amorphous and the transformation of α to γ has not occurred yet. After 160 h of milling, the authors decided to anneal the powders at 1100 °C for 1 h holding time under argon. The XRD data collected from annealed powder are presented in Figure 2. The microstructural analysis reveals that the annealed powders exhibit an FCC crystal structure. A similar trend of ball milling followed by high temperature annealing was reported by Haghir et al. [48].

**Figure 3:**
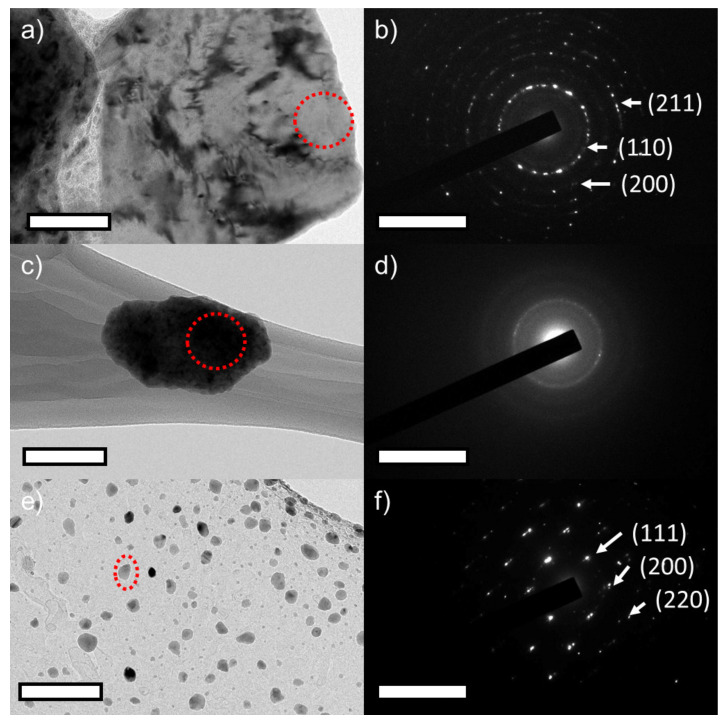
Bright field transmission electron microscopy (BF-TEM) of ball-milled Fe_(73−x)_-Cr_18_-Ni_9_-Si_x_, x = 3 wt % SS powders (**a**) after 5 h, (**c**) after 20 h, and (**e**) after 160 h followed by annealing respectively. (**b**,**d**,**f**) Selected area diffraction patterns collected from the red encircled areas in (**a**,**c**,**e**) respectively. The scale bar represents a length of 50 nm and the reciprocal space scale bar in the insets represents 10 1/nm.

FE-TEM was leveraged to confirm the XRD results—i.e., formation of α-phase at 5 h milling, amorphous phase at 20 h, and evolution of γ-phase after annealing—and the data are presented in Figure 3. Figure 3a,b shows the BF-TEM image of 5 h milled powder and the respective SAED pattern. The SAED pattern matches with that of the α-phase. This confirmed the formation of α-phase at 5 h of milling. Figure 3c,d shows the BF-TEM image of 20 h milled powder and its respective SAED pattern. The BF-TEM images confirm the particle refinement due to milling and its SAED pattern shows the diffused rings of amorphous phase as indicated by XRD results. Figure 3e,f shows the BF-TEM images of 160 h milled and annealed powder and its respective SAED pattern. The BF-TEM images confirm the particle refinement at 160 h and its SAED diffraction pattern confirms the γ-phase formation after annealing. These TEM investigations confirmed the XRD results.

### 3.2. Characterization of Spark Plasma Sintered Specimens

**Figure 4:**
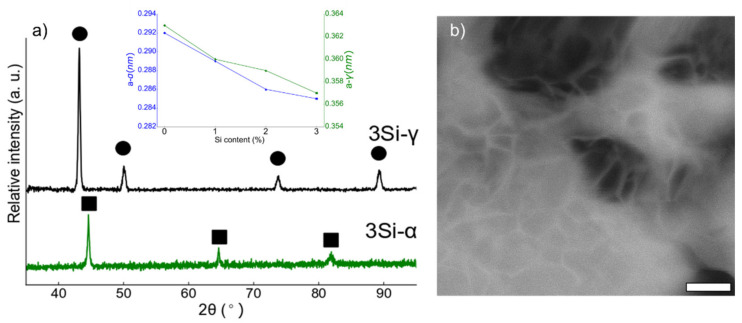
(**a**) The XRD spectra collected for ferritic (Fe_(82−x)_-Cr_18_-Si_x_, x = 3 wt %) and austenitic (Fe_(73−x)_-Cr_18_-Ni_9_-Si_x_, x = 3 wt %) SS spark plasma sintered specimens. Ferritic SS powders are represented as α and austenitic SS powders with γ. The square markings in (**a**) represent the BCC peaks and circles denote FCC peaks. The inset in (**a**) represents the estimated change in unit cell constant (after sintering) with the change in Si content. (**b**) Back scattered electron (BSE) image of 3 wt % Si, austenitic stainless-steel alloys after etching in 3% Nital solution (scale bar = 300 nm).

The alloys were sintered under optimized conditions of 1100 °C for 10 min, applied pressure of 60 MPa, and at a heating rate of 100 °C/min under vacuum. The XRD spectra collected from the spark plasma sintered ferritic (Fe_(82−x)_-Cr_18_-Si_x_, x = 3 wt %) and austenitic (Fe_(73−x)_-Cr_18_-Ni_9_-Si_x_, x = 3 wt %) specimens are presented in Figure 4a. Similar XRD spectra are collected from (Fe_(82−x)_-Cr_18_-Si_x_, x = 0, 1, and 2 wt %) austenitic (Fe_(73−x)_-Cr_18_-Ni_9_-Si_x_, x = 0, 1, and 2 wt %). The analysis of the XRD data confirmed the BCC and FCC crystal structures of ferritic and austenitic SS specimens, respectively. The inset in Figure 4a presents the change in the unit cell constant with the change in % Si content (after sintering). In both ferritic and austenitic SS systems, lattice constant decreases monotonically. Figure 4b is a back-scattered electron (BSE) image collected from 3 wt % Si, austenitic stainless steel alloy after etching in 3% Nital solution. The average grain size was estimated by measuring the grain sizes using built-in tools in SEM and was calculated to be 65.72 nm, which confirmed that nanocrystalline microstructure was preserved after the SPS process under the specified conditions.

**Figure 5:**
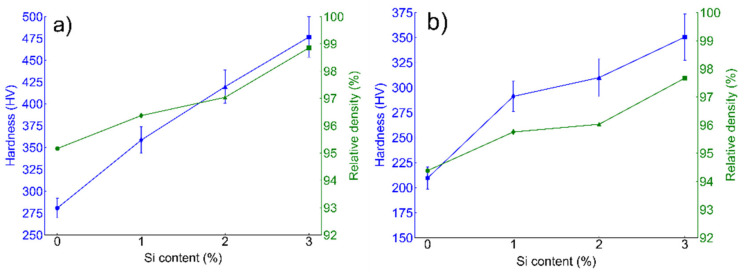
Micro-hardness and % relative density estimated from (**a**) ferritic (Fe_(82−x)_-Cr_18_-Si_x_, x = 0, 1, 2, and 3 wt %) and (**b**) austenitic (Fe_(73−x)_-Cr_18_-Ni_9_-Si_x_, x = 0, 1, 2, and 3 wt %) SS sintered specimens. Micro-hardness was estimated from at least 10 different indentations and the average is presented here. Both the ferritic and austenitic sintered systems indicate the increasing trend of density and hardness with the increase in wt % Si.

Micro-hardness and relative densification data calculated from both BCC (ferritic SS) and FCC (austenitic SS) are presented in Figure 5a,b respectively. Both BCC and FCC sintered alloys exhibited an increasing trend in relative densification with wt % Si. In the case of ferritic (Fe_(82−x)_-Cr_18_-Si_x_, x = 0, 1, 2, and 3 wt %) SS sintered specimens, the maximum densification achieved was lesser than 98% while in case of austenitic specimens (Fe_(73−x)_-Cr_18_-Ni_9_-Si_x_, x = 0, 1, 2, and 3 wt %), at the same SPS conditions, ~99% relative density was observed. Micro-hardness (HV) values show an increasing trend with the increase in % relative density. Starting with the α-BCC system, a minimum and maximum hardness was calculated at 209.6 ± 18.2 HV and 350.6 ± 11.2 HV for 0 wt % and 3 wt % Si, respectively. Similarly, for the γ-FCC system, minimum and maximum hardness was calculated at 280.8 ± 13.2 HV and 476.6 ± 15.2 HV for 0 wt % and 3 wt % Si, respectively.

### 3.3. Electrochemical Investigations

#### 3.3.1. Potentiodynamic Polarization (PDP)

**Figure 6:**
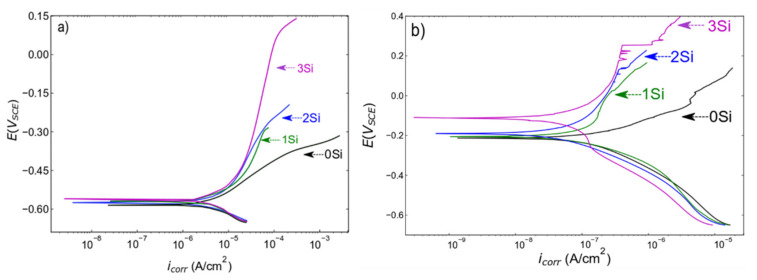
Potentiondynamic polarization scans collected from (**a**) ferritic (Fe_(82−x)_-Cr_18_-Si_x_, x = 0, 1, 2, and 3 wt %) and (**b**) austenitic (Fe_(73−x)_-Cr_18_-Ni_9_-Si_x_, x = 0, 1, 2, and 3 wt %) SS sintered specimens in deaerated 0.2 M NaCl solution with a scan rate of 0.2 mV/sec at room temperature. Deaerating was performed by purging nitrogen gas into the electrolyte for 30 min prior to testing. Each sample was tested at least three times at different locations but under similar conditions. The scans presented here are the representative of three scans.

The PDP scans collected from both ferritic (Fe _(82−x)_-Cr_18_-Si_x_, x = 0, 1, 2, and 3 wt %) and austenitic (Fe_(73−x)_-Cr_18_-Ni_9_-Si_x_, x = 0, 1, 2, and 3 wt %) SS sintered specimens in a deaerated 0.2 M NaCl solution at room temperature are presented in Figure 6a,b, respectively. The corrosion resistance of the sintered alloys measured in terms of series of kinetic parameters (pitting potential (*E_pit_*), passive current density (*i_pass_*), and corrosion potential (*E_corr_*) etc.) are presented in Table 1. To confirm the reproducibility of the data, PDP scans were collected at three different locations on the same sample under similar electrochemical conditions. Both ferritic and austenitic SS sintered alloys exhibited a similar trend of increase in corrosion resistance with the increase in wt % Si. Starting with ferritic SS sintered samples, Figure 6a and Table 1 indicates that there is no significant difference in *E_corr_*, but that *i_corr_* monotonically decreases with the increase in wt % Si, indicating the higher propensity of the 0 wt % Si sample to corrosion. Furthermore, to estimate *i_pass_*, a potential at −0.55 V_SCE_ (in the passive region) was selected and the data indicate that passive current density is not changing with wt % change in Si. However, the electrochemical performance significantly changes; i.e., the width of passive region increases. Figure 6a shows that the 0 wt % Si sample does not exhibit a clear passive region while the 3 wt % Si sample exhibits a wider passive region. This trend is reported in terms of *E_pit_* values in Table 1; since the 0 wt % Si sample is not passivating, *E_pit_* is indicated as N/A; and for the 3 wt % Si, *E_pit_* is calculated at 0.128 V_SCE._ The difference in *E_pit_* values from the 0 wt % Si to the 3 wt % Si sample clearly indicate an increase in corrosion resistance due to the formation of more protective passive film with an increase in Si content (reasons are presented in the Discussion section). Similar analysis of kinetic parameters obtained from the austenitic SS sintered specimens is represented in Table 1. The analysis of austenitic SS sintered specimens showed that the 0 wt % Si exhibits the lowest *E_corr_* and highest *i_corr_* estimated at −0.231.6 V_SCE_ and 0.886 pA/cm^2^ respectively. The sample with 3 wt % Si exhibits the highest *E_corr_* and lowest *i_corr_* values estimated at −104.4 V_SCE_ and 739.2 pA/cm^2^ respectively. The visual comparison of PDP curves indicates that the 0 wt % Si sample does not exhibit a clear passive region and the 3 wt % Si sample has a wider passive region. The comparative analysis of *E_pit_* values indicates a monotonic increase in corrosion resistance with an increase in Si content. To conclude, increase in the width of a passive region in both systems with an increase in Si content is directly related to the improvement in electrochemical performance. The electrochemical performance comparison between ferritic and austenitic SSs is presented in the Discussion section.

#### 3.3.2. Linear Polarization Resistance (LPR)

**Figure 7:**
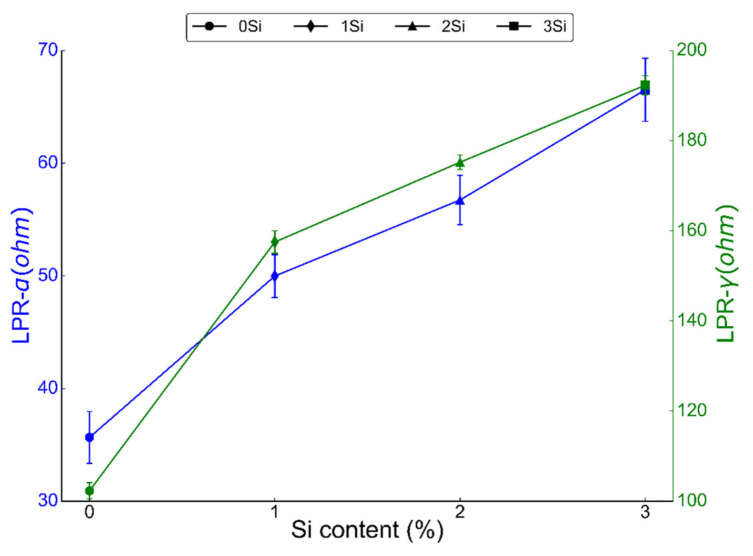
Linear polarization resistance (LPR) from both ferritic (Fe_(82−x)_-Cr_18_-Si_x_, x = 0, 1, 2, and 3 wt %) and austenitic (Fe_(73−x)_-Cr_18_-Ni_9_-Si_x_, x = 0, 1, 2, and 3 wt %) SS sintered specimens in deaerated 0.2 M NaCl solution at room temperature with a scan rate of 0.3 mV/sec. Each sample was tested at least three times at different locations but under similar conditions, and the LPR presented here is the representative of three scans.

LPR was conducted by sweeping the potential from −0.02 to 0.02 V at a scan rate of 0.3 mV/sec in 0.2 M NaCl solution at room temperature. LPR is a direct measure of the passive film’s resistance to corrosion as higher polarization resistance indicates the higher strength of the passive film in a corroding environment [49]. LPR was calculated for both ferritic (Fe_(82−x)_-Cr_18_-Si_x_, x = 0, 1, 2, and 3 wt %) and austenitic (Fe_(73−x)_-Cr_18_-Ni_9_-Si_x_, x = 0, 1, 2, and 3 wt %) sintered specimens (Figure 7). Both alloys showed an increasing trend in LPR with the increase in Si content of the alloys. Both ferritic and austenitic samples without any Si (0 wt % Si) exhibited the lowest LPR, i.e., 35.66 ± 2.3 and 102.3 ± 1.8 ohm, respectively. On the other hand, LPR of 3 wt % Si in ferritic and austenitic samples was found to be 66.51 ± 2.1 and 192.3 ± 2.1 ohm, respectively. LPR data further confirm that the addition of Si in both ferritic and austenitic SS sintered specimens increased the corrosion resistance of these alloys.

#### 3.3.3. Electrochemical Impedance Spectroscopy (EIS)

**Figure 8:**
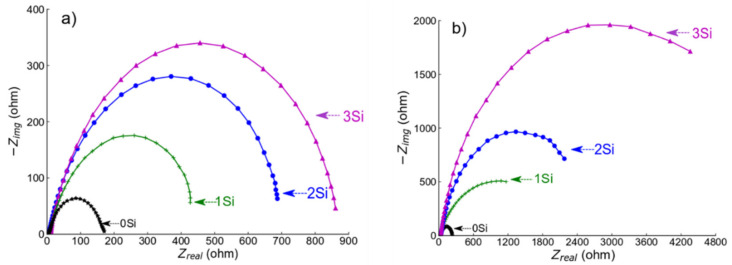
Electrochemical impedance spectroscopy (EIS) spectra collected from (**a**,**b**) ferritic (Fe_(82−x)_-Cr_18_-Si_x_, x = 0, 1, 2, and 3 wt %) and austenitic (Fe_(73−x)_-Cr_18_-Ni_9_-Si_x_, x = 0, 1, 2, and 3 wt %) SS sintered specimens in deaerated 0.2 M NaCl solution at room temperature. Deaeration was performed by purging nitrogen into the electrolyte for 30 min prior to testing. EIS scans were collected at a passive potential −0.55 VSCE (for ferritic SS) and −0.05 VSCE (for austenitic SS).

**Figure 9:**
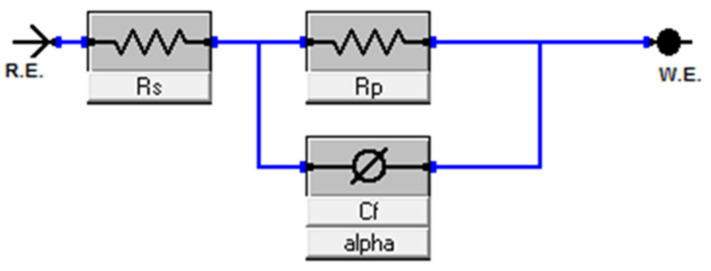
Electrical equivalent circuit (EEC) model used for fitting the experimentally collected EIS data. In the model, Rs represents the solution/electrolyte resistance, while Rp is the polarization resistance of the passive film and Cf is the constant phase element. R.E. represents the reference electrode (here, Pt wire) and W.E. denotes the working electrodes (sintered samples).

EIS experiments were performed at −0.55 V_SCE_ (for ferritic SS) and −0.05 V_SCE_ (for austenitic SS) in deaerated 0.2 M NaCl at room temperature. The potential was selected from the PDP data in Figure 6a,b, respectively, as the potential lies in the passive region for all the alloys. Before conducting the test, a passive film was formed for about 30 min. EIS spectra (Nyquist plots) obtained from both ferritic (Fe_(82−x)_-Cr_18_-Si_x_, x = 0, 1, 2, and 3 wt %) austenitic (Fe_(73−x)_-Cr_18_-Ni_9_-Si_x_, x = 0, 1, 2, and 3 wt %) are presented in Figure 8a,b, respectively. The diameter of semi-circles in the Nyquist plot is directly related to the polarization resistance of the passive film [50] and the EIS data presented in Figure 8 indicate the monotonic increase in diameter with the increase in wt % Si. Since EIS curves exhibited only one time constant, the electrical equivalent circuit (EEC), shown in Figure 9, was used for quantitative study of the spectrum, and the results showed a good quality fitting with this model. In the equivalent circuit, a constant phase element (CPE) was used instead of capacitance because many times the measured capacitance is not ideal. In the model, *Rs* represents the solution/electrolyte resistance, while *Rp* is the polarization resistance of the passive film. The data collected after fitting the experimental EIS data with the EEC model are presented in Table 2. The comparative analysis of *Rp* values estimated after the EEC model fit indicates a monotonic increase with wt % Si in both ferritic and austenitic SS samples.

## 4. Discussion

### 4.1. Microstructure and Mechanical Characterization

The mechanical properties—especially corrosion resistance—of powder metallurgy (PM) steels is strongly dependent on the density of the sintered alloys. Many sintering parameters such as sintering temperature [13], holding time [51], heating rate [52], sintering environment [53], and applied pressure are crucial in obtaining a dense PM material. In a previous study [16], the authors systemically varied sintering temperature, holding time, heating rate, and applied pressure to obtain the maximum densification. Indeed, higher temperature, time, pressure, and slower heating yields higher densification and optimized sintering conditions (1100 °C for 15 min, applied pressure of 60 MPa and a heating rate of 100 °C/min under vacuum) as presented in detail elsewhere [16]. Both ferritic (Fe_(82−x)_-Cr_18_-Si_x_, x = 0, 1, 2, and 3 wt %) and austenitic (Fe_(73−x)_-Cr_18_-Ni_9_-Si_x_, x = 0, 1, 2, and 3 wt %) SS are synthesized under similar ball milling conditions (except austenitic SS powders milled up to 160 h followed by annealing) and sintered under the same conditions. The examination of the XRD spectrum of austenitic stainless steel powders milled for 160 h revealed a characteristic amorphous spectrum. Ferritic powders also showed a similar trend for up to 50 h of milling; however, continued milling up to 85–100 h resulted in a crystallized alpha phase [40]. On the other hand, austenitic powders remained amorphous up to 160 h of milling; subsequently, an annealing treatment was carried out at 1100 °C/1 h under argon atmosphere. X-Ray diffraction (XRD) of the annealed powder confirmed the formation of crystallized austenite (γ)-phase (Figure 2). The present study was aimed at underscoring the importance of Si content on the evolution of particle size during ball milling and consequently on the densification of PM SS parts. Si played an important role in grain refinement during ball milling as reported elsewhere [40]. Similarly, Yousefi et al. [54] observed that the presence of Si in Fe-Cr alloys makes the solid solution particles harder and that results in decreased grain size. The intrinsic hardness of the particles promotes the fragmentation of particles during ball milling and so consequently yields particle refinement [55]. Obviously, milling time is an important parameter affecting the final particle size of the alloyed powders as higher milling time yields smaller particles [28]. As reported in Section 3.1, austenitic SS powders were milled for 160 h in comparison to ferritic SS powders (milled for 100 h), and thus exhibited much finer particle size. Powder particle size can have a direct effect on the final densification and subsequent mechanical properties of the sintered specimen. Ertugrul et al. [56] highlighted the benefits of using smaller-size powder particles in obtaining higher % densification via conventional sintering. On the other hand, Diouf et al. [47] favored the SPS technique to sinter copper powder with varying particle sizes and reported an opposite trend; bigger particles yield higher densification. The comparative analysis of relative density data reported in Figure 5a,b for ferritic SS (Fe_(82−x)_-Cr_18_-Si_x_, x = 3 wt %) and austenitic SS (Fe_(73−x)_-Cr_18_-Ni_9_-Si_x_, x = 3 wt %) indicates that austenitic alloy exhibited the higher density. A possible reason for the monotonic increase in relative density in both ferritic and austenitic SS can be the presence of Si in these alloys. Many studies [16,38,57] have reported that the presence of Si increased the overall density of alloys. Tsai et al. [38] reported that the presence of Si in the powder mix developed a ‘pseudo-peritectic’ reaction resulting in the improvement of overall density. Toor et al. [16] suggested that, due to the lower melting temperature of Si and temperature confinement in SPS technique, localized melting can occur which promotes densification.

### 4.2. Electrochemical Mechanism in PM Alloys

**Figure 10:**
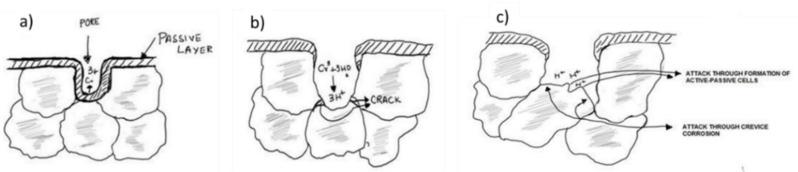
Schematic explaining the corrosion mechanism of powder metallurgy stainless steels by leveraging the analogy of crevice corrosion as explained by Otero et al. [12]. (**a**) The first interaction of corrosive electrolyte with the intrinsic porosity/crevice due to sintering. (**b**) Breakdown of the passive layer due to excessive corrosion inside the porosity/crevice and establishment of differential airing cavities. (**c**) Release of excessive protons inside the crevice makes the electrolyte more corrosive and the differential active–passive cell that is established promotes corrosion. This figure is redrawn from the work by Otero et al. [12].

The comparison of the data presented above in Figure 6 (PDP scans), Figure 7 (LPR data), and Figure 8 (EIS spectra) highlights that the corrosion resistance of both ferritic and austenitic SS sintered specimens increased with an increase in Si content. Since corrosion resistance of PM parts is directly influenced by their densification as reported by many authors [17,45,51,52], one possible reason for the improvement in corrosion resistance properties is increased relative density of the higher wt % Si alloys as reported elsewhere [53,54]. The % of porosity in the sintered SS parts can directly influence the protectiveness of the oxide film developed on such materials. Bautista et al. [56] reported that the irregularities introduced due to the presence of surface porosity in the PM SS can seriously affect the formation of a smooth passive layer. Simply put, the passive layer formed on the surface of the samples with higher porosity is intrinsically less protective. In another study [55], it was reported that the presence of open surface porosities in sintered PM alloys exponentially increases the exposed surface area to electrolytic attack. The authors reported that the active area exposed corrosive electrolytes is 10- to 100-times greater than the geometrical area. Furthermore, Otero et al. [12] presented the analogy of crevice corrosion to explain the higher corrosion rates in sintered stainless steels. The micro-pores present on the surface of the sintered alloys act as crevices and are more prone to electrolytic attacks. The geometry, size, % fraction of pores, and the nature of electrolyte determines the severity of corrosion in such materials. Corrosion starts in the crevices/pores and become more widespread, progressively moving toward the interior of the material and becoming more aggressive over time due to the localized changes in the electrolyte concentration inside the pores. The formation of differential aeration cavities and differential proton concentration inside the pores/crevices makes the localized electrolyte more corrosive. Simply put, all the oxygen inside the crevices is consumed while the sample surface can have immediate access to oxygen. This differential concentration establishes a local active–passive cells, causing an exponentially higher corrosion rate inside the crevices. Figure 10 schematically explains the corrosion mechanism in PM stainless steels using the analogy of crevice corrosion as leveraged by Otero et al. [12]. The other possible reason for improvement in the corrosion resistance of these alloys is the formation of Si incorporated passive film in these alloys. It is reported elsewhere [37,38,56] that the presence of Si improves the passive film quality due to the enrichment and development of Si-oxide in the passive film. Robin et al. [57] studied the composition of passive film formed on two different austenitic SSs with varying Si contents via X-ray photoelectron spectroscopy (XPS) and confirmed the presence of Si in the passive layer. Similarly, Tsai et al. [38] confirmed the beneficial effect of Si on the improvement in corrosion resistance due to the Si enrichment of the passive layer. These studies [37,38,58] confirmed that Si did indeed affect the overall quality of the passive layer formed on SSs.

Table 3 shows the comparison between the corrosion performances of the developed alloys with the ones reported in the literature (with different alloying additions). Cabral et al. [59] sintered pre-alloyed AISI 409 ferritic stainless steel powders with various % of boron content in order to increase their densification for better corrosion performance. A conventional sintering technique was followed and the conditions were set as follows: sintering temperature = 1150 °C, holding time = 60 min, and compaction pressure = 700 MPa under hydrogen atmosphere. The authors reported an increase in % densification with the increase in boron content [59,60] owing to a liquid phase sintering process. The maximum densification achieved was 93.7% for 1.2 wt % boron content. They also found some boron carbide formation at the grain boundaries. They conducted LPR studies in 0.5 M NaCl solution and the results are compared with this study in Table 3. As shown in the table, the introduction of 0.8 % boron significantly increased the LPR but further increase resulted in the degradation of electrochemical performance. Despite a monotonic increase in % of densification with the increase in boron content, the decrease in LPR values suggests that boron is detrimental to the overall corrosion performance and this is probably due to boron carbide precipitation at the grain boundaries when boron wt % is increased. On the other hand, in our methodology, the addition of Si content results in monotonic increase in LPR values as well as % densification. This indicates that the importance of the methodology employed in this study (ball milling followed by SPS) is an effective technique to improve the electrochemical performance of sintered steels.

### 4.3. Comparison of Ferritic SSs with Austenitic SSs

**Figure 11:**
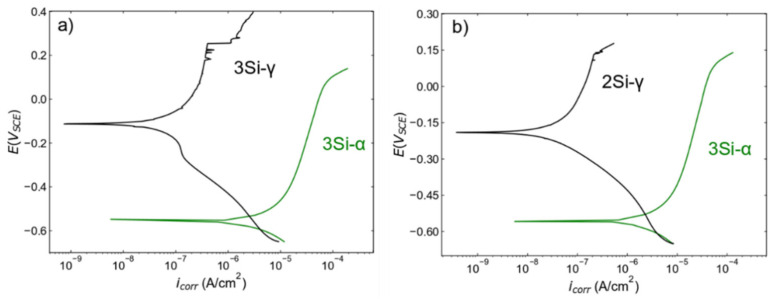
Comparative analysis of PDP scans collected from (**a**) ferritic (Fe_(82−x)_-Cr_18_-Si_x_, x = 3 wt %) and austenitic (Fe_(73−x)_-Cr=-Ni_9_-Si_x_, x = 3 wt %) and (**b**) ferritic (Fe_(82−x)_-Cr_18_-Si_x_, x = 3 wt %) and austenitic (Fe_(73−x)_-Cr_18_-Ni_9_-Si_x_, x = 2 wt %) stainless steel sintered specimens. These figures are redrawn from Figure 4a,b for comparison purposes.

The comparative analysis of ferritic SS (Fe_(82−x)_-Cr_18_-Si_x_, x = 3 wt %) and austenitic (Fe_(73−x)_-Cr_18_-Ni_9_-Si_x_, x = 3 wt %) SS samples indicates that austenitic SSs are more corrosion resistant than ferritic SSs (Figure 11a). It is important to note that the density of ferritic SS (Fe_(82−x)_-Cr_18_-Si_x_, x = 3 wt %) is around 98% and austenitic (Fe_(73−x)_-Cr_18_-Ni_9_-Si_x_, x = 3 wt %) SS is around 99%, but the corrosion response is dramatically different. Therefore, it is safe to conclude that the dramatic increase in corrosion resistance is not due to the relative densification. For better comparison, a PDP plot for ferritic SS (Fe _(82−x)_-Cr_18_-Si_x_, x = 3 wt %, density = 98%) is plotted against austenitic SS (Fe_(73−x)_-Cr_18_-Ni_9_-Si_x_, x = 2 wt. %, density~99) Figure 11b. This comparative analysis confirms that the increase in corrosion resistance is due to the presence of Ni in austenitic SS. Azuma et al. [61] performed a systemic study on the effect of Ni addition on the corrosion behavior of ferritic SSs in seawater and reported an increase in corrosion resistance. Ujiro et al. [62] reported that ferritic SS with a similar Cr content to austenitic SS is less corrosion resistant in saline water. Similar comparison between ferritic and austenitic SSs was conducted by Loto et al. [63] to confirm the inferior corrosion resistance of ferritic SS in chloride solutions. The comparative analysis (Figure 11b) and the literature [63,64] highlight the positive role of Ni content on the passive layer formation and stability in chloride solutions. Abreu et al. [64] leveraged EIS in conjunction with the XPS technique to study the change in thickness of passive layer formation in AISI 430 and AISI 304 SS with varying Ni contents. The authors reported the higher Ni content in the austenitic SS manifests in thinner and more protective passive film in comparison to ferritic SS with lower Ni content. Loto et al. [63] also highlighted the increase in stability of passive film in austenitic SS. These results further solidify the concept that Ni promotes the formation of a more protective passive film—and thus better corrosion resistance—exhibited by austenitic stainless steels.

## 5. Conclusions

Nanostructured ferritic (Fe_(82−x)_-Cr_18_-Si_x_, x = 0, 1, 2, and 3 wt %) and austenitic (Fe_(73−x)_-Cr_18_-Ni_9_-Si_x_, x = 0, 1, 2, and 3 wt %) stainless steel powder alloys were prepared by mechanical alloying (MA). Various techniques such as X-ray diffraction (XRD), scanning electron microscopy (SEM), and field emission transmission electron microscopy (FE-TEM) were leveraged for microstructural characterizations. Ferritic powders exhibiting a body centered cubic (BCC) structure were obtained after 100 h of milling while austenitic powders were milled for 160 h followed by annealing at 1100 °C for 60 min in an argon atmosphere. The particle size after ball milling was estimated from low magnification FE-TEM images. Ferritic (Fe_(82−x)_-Cr_18_-Si_x_, x = 3 wt %) SS powders have an average particle size of 65 ± 5 nm and austenitic (Fe_(73−x)_-Cr_18_-Ni_9_-Si_x_, x = 3 wt %) powders average 18 ± 3 nm. Spark plasma sintering (SPS) was performed at 1100 °C for 10 min under an applied pressure of 60 MPa and at a heating rate of 100 °C/min under vacuum. XRD data confirm the BCC and face centered cubic (FCC) crystal structure of ferritic and austenitic SSs, respectively. The percentage of densification was estimated by Archimedes’ principle and the data indicate that both alloy systems exhibit an increasing trend of densification with the increase in wt % Si content. In both ferritic and austenitic systems maximum densifications of ~98% and ~99%, respectively, were observed for 3 wt % Si. A similar trend was observed for micro-hardness: a 3 wt % system exhibited the highest micro-hardness. The comparative analysis of corrosion kinetic parameters for the ferritic system (Fe_(82−x)_-Cr_18_-Si_x_, x = 0, 1, 2, and 3 wt %) showed that there was no significant difference in corrosion potential (*E_corr_*) values (i.e., min = −511.8 mV and max = −454.5 mV); however, pitting potential was significantly improved for 3 wt % Si alloys. Visual comparison of PDP scans shows that a 0 wt % Si system does not exhibit a clear passive region while a 3 wt % Si system exhibited a wide passive region. The maximum *E_pit_* was estimated to be 125 mV for a 3 wt % Si system. A similar trend of kinetic parameter variation was observed for an austenitic system at 0 wt %. Si has no clear passive region and 3 wt % Si exhibited a wide passive region. Maximum *E_pit_* was estimated to be 259 mV. LPR values indicated a monotonic increase with the wt % Si content in both alloy systems. An electrical equivalent circuit (EEC) with a constant phase element was used to fit the EIS data, and the results showed an increase in electrochemical impedance performance of the alloys with an increase in Si content of the alloy systems.

## Figures and Tables

**Table 1:** Kinetic parameters estimated from potentiodynamic polarization scans collected from both ferritic (Fe_(82−x)_-Cr_18_-Si_x_, x = 0, 1, 2, and 3 wt %) and austenitic (Fe(73−x)-Cr18-Ni9-Six, x = 0, 1, 2, and 3 wt %) SS sintered specimens in 0.2 M NaCl solution at room temperature. To estimate the passive current density (*i_pass_*), passive potential at −0.55 and −0.05 VSCE was selected for ferritic and austenitic SS respectively. In the case of 0 wt % Si for both ferritic and austenitic SS, *E_pit_* and *i_pit_* are not available (N/A).

Material (wt % Si)	Ferritic SS (α)				Austenitic SS (γ)			
	$E_{corr}$ (mV)	$i_{corr}$ (µA)	$E_{pit}$ (mV)	$i_{pit}$ (mA/cm^2^)	$E_{corr}$ (mV)	$i_{corr}$ (pA/cm^2^)	$E_{pit}$ (mV)	$i_{pit}$ (µA/cm^2^)
0	−511.8	98.42	N/A	N/A	−231.6	0.886	N/A	N/A
1	−485.8	86.66	−220.1	822.2	−233	0.773	−18.2	279.2
2	−460	63.63	−164.1	876.3	−173.5	565.2	134.2	146.8
3	−454.5	10.08	125.2	889.2	−104.4	739.2	259.6	112.2

**Table 2:** Parameters estimated from EIS curve fitting with the EEC model presented in Figure 7 for both ferritic (Fe_(82−x)_-Cr_18_-Si_x_, x = 0, 1, 2, and 3 wt %) and austenitic (Fe_(73−x)_-Cr_18_-Ni_9_-Si_x_, x = 0, 1, 2, and 3 wt %) SS sintered specimens.

Material (wt % Si)	Ferritic SS (α)			Austenitic SS (γ)		
	$R_{p}$ (Ω)	$R_{s}$ (Ω)	CPE (µF/cm^2^)	$R_{p}$ (Ω)	$R_{s}$ (Ω)	CPE (µF/cm^2^)
0	171.1	35.85	0.835	214.5	34.18	1.99
1	424.2	36.22	0.320	1.87e3	33.81	1.67
2	722.7	37.34	0.260	2.16e3	28.46	1.64
3	876.5	40.16	0.238	564e3	21.07	1.10

**Table 3:** Comparative analysis of linear polarization resistance (LPR) values estimated in this study with the values calculated by Cabral et al. [59]. The authors sintered pre-alloyed AISI 409 Nb stabilized powders with various % of boron content. The nomenclature defined by the authors indicate the % of boron added to the pre-alloyed powders. The authors used 0.5 M NaCl solution to perform the electrochemical investigations.

Sample Name	LPR (Ω-cm^2^)	Sample Name	LPR (Ω-cm^2^) [59]
γ–0Si	511.5	Fe409	529.39
γ–1Si	787.5	Fe0.8B	961.74
γ–2Si	876.2	Fe1.0B	584.14
γ–3Si	961.5	Fe1.2B	583.02

## Data Availability

The data presented in this study are available on request from the corresponding author.
